# Stimulated bacterioplankton growth and selection for certain bacterial taxa in the vicinity of the ctenophore *Mnemiopsis leidyi*

**DOI:** 10.3389/fmicb.2012.00302

**Published:** 2012-08-16

**Authors:** Julie Dinasquet, Lena Granhag, Lasse Riemann

**Affiliations:** ^1^Department of Natural Sciences, Linnaeus UniversityKalmar, Sweden; ^2^Marine Biological Section, University of CopenhagenHelsingør, Denmark; ^3^Department of Marine Ecology-Kristineberg, University of GothenburgGothenburg, Sweden; ^4^Department of Shipping and Marine Technology, Chalmers University of TechnologyGothenburg, Sweden

**Keywords:** *Mnemiopsis leidyi*, bacterioplankton, ctenophore, bacterial community composition

## Abstract

Episodic blooms of voracious gelatinous zooplankton, such as the ctenophore *Mnemiopsis leidyi*, affect pools of inorganic nutrients and dissolved organic carbon by intensive grazing activities and mucus release. This will potentially influence bacterioplankton activity and community composition, at least at local scales; however, available studies on this are scarce. In the present study we examined effects of *M. leidyi* on bacterioplankton growth and composition in incubation experiments. Moreover, we examined community composition of bacteria associated with the surface and gut of *M. leidyi*. High release of ammonium and high bacterial growth was observed in the treatments with *M. leidyi* relative to controls. Deep 454 pyrosequencing of 16 S rRNA genes showed specific bacterial communities in treatments with *M. leidyi* as well as specific communities associated with *M. leidyi* tissue and gut. In particular, members of Flavobacteriaceae were associated with *M. leidyi*. Our study shows that *M. leidyi* influences bacterioplankton activity and community composition in the vicinity of the jellyfish. In particular during temporary aggregations of jellyfish, these local zones of high bacterial growth may contribute significantly to the spatial heterogeneity of bacterioplankton activity and community composition in the sea.

## Introduction

In the past decade, the lobate ctenophore *Mnemiopsis leidyi*, which is native to the American east coast (Kremer, 1994), has spread to the Black and Caspian Seas (Finenko et al., 2006), the Mediterranean Sea (Shiganova et al., 2001), and more recently to the North Sea and the southern part of the Baltic Sea (e.g., Tendal et al., 2007). While it is well documented that this voracious predator (Colin et al., 2010) may graze upon a variety of plankton taxa (Granhag et al., 2011) and elicit cascading food web effects (Dinasquet et al., 2012), bottom-up effects associated with presence and activity of *M. leidyi* have received less attention.

Jellyfish may stimulate bacterioplankton growth by direct release of nutrients, ammonia in particular (Kremer, 1977), from tissue, mucus secretion, excretion, and sloppy feeding (reviewed in Pitt et al., 2009). High bacterial growth has been observed in the vicinity of decaying jellyfish (Titelman et al., 2006; Tinta et al., 2010); however, even more importantly, growth of bacterioplankton surrounding live jellyfish may be stimulated by the release of nutrients and bioavailable carbon (Condon et al., 2011). For instance, elevated bacterial growth was observed in a 0.5 m diameter zone around an *Aurelia aurita* medusa (Hansson and Norrman, 1995). Given that jellyfish have the potential to affect bacterioplankton growth, it is conceivable that they also affect bacterioplankton community composition. Release of bioavailable carbon would presumably select for specific bacterial groups, as recently observed using fluorescence *in situ* hybridization (Condon et al., 2011), but in addition release of specific bacterial taxa from colonized jellyfish tissue or from the jellyfish gut could affect composition of bacterioplankton in close proximity to the jellyfish, assuming that tissue or gut harbor specific communities. To what extent jellyfish are colonized by bacteria is to our knowledge not known; however, small crustaceans like copepods and cladocerans can host high densities of bacteria (Tang, 2005; Tang et al., 2010). Jellyfish, in contrast to crustacean zooplankton, do not change exoskeletons or molt during their life, but instead the same jellyfish surface decrease and increase during starvation and growth. Hence, it is conceivable that a microbiota inhabits surfaces of jellyfish. Consequently, as reported for e.g., non-gelatinous zooplankton (Grossart et al., 2010), fecal pellets (Jacobsen and Azam, 1984), or model aggregates (Kiørboe et al., 2003), a continuous exchange of bacteria between jellyfish surfaces and the surrounding water is conceivable.

In the present study we applied small-scale incubations with specimens of *M. leidyi* to examine effects on bacterioplankton growth and composition. Moreover, we examined community composition of bacteria associated with the surface and gut of *M. leidyi*. Specifically, we aimed to test the hypotheses that (1) presence and feeding activity of *M. leidyi* affect bacterioplankton community structure and stimulate specific bacteria taxa in the vicinity of the jellyfish, and (2) bacteria attached to *M. leidyi* tissue or in the gut differ from those in the surrounding water.

## Materials and methods

### Experimental set-up

The experiment was initiated on 27 October 2010 at the Sven Lovén Centre for Marine Sciences in the Gullmar fjord at the west coast of Sweden (58° 15′N, 11° 27′E). At this time of the year only, *M. leidyi* is consistently present and performs local blooms (Friis-Møller, Tiselius; pers. comm.). Surface water, salinity 32, obtained from the fjord was 0.2 μm filtered (Supor filter, Pall), amended with 10% v/v 0.65 μm filtered (Millipore) fjord water (i.e., a bacterial inoculum), and 100 ml volumes were distributed in ten 100 mL acid-washed flasks, and manipulated in the following manner: three “control” treatments contained (1) water and inoculum as described above, (2) water and inoculum but with *M. leidyi* dipped into the water for 1 min to examine the potential immediate release of loosely associated bacteria from the *M. leidyi* tissue, and (3) water and inoculum with added copepods to examine potential release of bacteria from the copepods used as food items. Two additional treatments were amended with starved and fed *M. leidyi*, respectively. All treatments, in duplicates, were incubated for 41 h at 17°C under a 16:8 light:dark (h) cycle. Nutrients and bacterial community composition were measured at the start and end of the incubation, while bacterial abundance was measured six times (after 2, 13, 26, 37, and 41 h) during the course of the experiment. Nutrients and bacterial abundance were measured in the replicate flask. Since community composition was analyzed in only one replicate for each treatment, pseudoreplicates were used for ANOVA analysis of *M. leidyi* treatments (starved, fed, gut, and tissue) vs. controls.

### Ctenophore and copepod treatments

The *M. leidyi* specimens were collected on 20 October 2010 with buckets from the surface in the Gullmar fjord and acclimatized in the laboratory for a week before the experiment. They were fed mixed zooplankton collected daily from the fjord using a 90 μm net. One day prior to the experiment feeding of ctenophores ceased, either without further addition of food (for starved *M. leidyi)* or with a final feeding with a few copepods (adult *Acartia sp.*) just before experiment start (for fed *M. leidyi*). At the beginning of the experiment, it was visually confirmed that *M. leidyi* specimens had empty guts (starved) or contained 3–4 visible copepods (fed). Each flask contained one ctenophore with an oral–aboral length of 10 mm. The *M. leidyi* specimens were continuously monitored. They were swimming and appeared in good condition throughout the whole experiment. The fed *M. leidyi* digested the copepods in their guts within ~2 h.

Tissue (~2 × 2mm) from one of the dipped *M. leidyi* was aseptically dissected under a stereomicroscope from the ctenophore surface (mesoglea) and gut (pharynx) and stored frozen in sterile Eppendorf tubes at −80°C until DNA extraction. The specimen was treated in the same way as the starved animals and was not fed for 24 h prior to the dissection. A starved specimen was chosen in order not to include microbiota associated with prey in the gut analysis.

In the zooplankton incubation, three copepods (adult *Acartia* sp.) were added per flask and they remained in the flask throughout the incubation. The copepods were from the same zooplankton tow as was used in the final feeding of *M. leidyi* before the experiment.

### Measurements of inorganic nutrients and bacterial abundance

At termination of the experiment 10 mL water from each treatment were 0.45 μm filtered (Millipore) and frozen at −20°C. The samples were thawed and analyzed for ammonium, nitrate, and nitrite as well as phosphate with a TRAACS 2000 (Bran + Luebbe). For nutrients one-factor ANOVAs were conducted to test for differences between control and *M. leidyi* treatments. Samples (1.5 mL) for bacterial enumeration were fixed with EM grade glutaraldehyde (Sigma; 1% final conc.), frozen in liquid N_2_ and stored at −80°C. Cells were stained with SYTO 13 (Molecular Probes) and counted on a FACSCalibur flow cytometer (Becton Dickinson) according to Gasol and del Giorgio (2000). Fluorescent beads (True counts, Becton Dickinson) were used to calibrate the flow rate. Replicate counts of the same sample generally varied less than 5%. For bacterial abundance one-factor ANOVA was used to test for differences between the five treatments (pooling data from time 37 and 41) and Student-Newman-Keuls (SNK) post hoc test to determine between which treatments differences occurred. A Type I error rate of 0.05 was used.

### Bacterial community composition

Bacterial community composition was analyzed in a total of eight samples: six water samples (one replicate from the initial water and from each treatment after incubation), one *M. leidyi* tissue sample, and one *M. leidyi* gut sample. Water samples (80 mL) were filtered onto 0.2 μm sterivex filters (Millipore), which were then frozen at −80°C in 1 mL sucrose lysis buffer (20% sucrose, 50 mM EDTA, 50 mM TrisHCl, pH = 8). DNA was extracted from the sterivex filters using an enzyme/phenol-chloroform protocol (Riemann et al., 2000) but with a 30-min lysozyme digestion at 37°C and an overnight proteinase K digestion (20 mg ml^−1^ final conc.) at 55°C (Boström et al., 2004). DNA from *M. leidyi* tissue and gut samples was extracted using the EZNA tissue DNA kit (Omega Bio-Tek). DNA was quantified using Nanodrop (Thermo Scientific). Bacterial 16 S rRNA genes were PCR amplified using puReTaq Ready-To-Go PCR beads (GE Healthcare), 0.06 ng DNA μl^−1^, and primers 341F (5′-CCT ACG GGN GGC WGC AG-3′) and 805R (5′-GAC TAC HVG GGT ATC TAA TCC-3′). The amplification was run in two steps: a first step with the regular primers (20 cycles), followed by re-amplification of 1 μl product using the same primers complemented with 454-adapters and sample-specific barcodes (five cycles; Berry et al., 2011). For each sample, triplicate PCR products from independent runs were pooled prior to purification (Agencourt AMPure XP kit, Beckman Coulter) and quantification (PicoGreen, Molecular Probes). The samples were mixed in equimolar amounts and sequenced from the reverse primer direction using Roche/454 GS FLX Titanium technology (National High-throughput DNA Sequencing Centre, University of Copenhagen).

### Phylogenetic analysis

Sequences were analyzed and processed using the Quantitative Insights Into Microbial Ecology software (QIIME v1.4; Caporaso et al., 2010b) with default settings, excluding sequences <350 bp or >450 bp. Flowgrams were denoised directly in the pipeline (Reeder and Knight, 2010). All singletons were removed. Sequences were then clustered into operational taxonomic units (OTUs) at 97% pairwise identity using the seed-based Uclust algorithm, and representative sequences from each OTU aligned to the Greengenes imputed core reference alignment (DeSantis et al., 2006) (http://greengenes.lbl.gov) using PyNAST (Caporaso et al., 2010a). Chimeras were removed using Chimera Slayer (Haas et al., 2011). Taxonomy assignments were made using the ribosomal database project (RDP) classifier (Wang et al., 2007). Sequences have been deposited in the CAMERA database (Community Cyberinfracstructure for advanced microbial ecology research and analysis; http://camera.calit2.net/index.shtm) under accession number CAM_P_0000918. The phylogenetic similarity between samples was determined with the unweighted pair group method using an arithmetic (UPGMA) mean tree calculated from the jack-knife-weighted UniFrac distance matrix within QIIME. The matrix was calculated with randomly picked OTUs (normalized to 1043 sequences per sample to accommodate for the lowest number of sequences found in a sample). The random subsampling was done within QIIME. In order to present a succinct overview of the main taxa in the samples, OTUs summing to >100 sequences across all samples were used for further log 10 + 1 transformation and visualization in a heatmap. The heatmap was made using the CIMminer tool for clustered image maps (http://discover.nci.nih.gov/).

## Results and discussion

### Nutrient concentrations and bacterial growth

Release of ammonium in the *M. leidyi* treatments (starved and fed) was higher than in control treatments (one factor ANOVA *F*_(1, 8)_ = 89, *p* = 0.0001; Table 1). The ammonium release was more variable in fed *M. leidyi*, which may be due to variations in digestion time and start of starvation between specimens. For the starved *M. leidyi*, ammonium release within the two replicates had similar values. The ammonium release was expected to be higher in starved animals as ammonium is an end product during starvation, which initiates within few hours without food (Kremer and Reeve, 1989). This excretion is dependent on the feeding history of the ctenophores (Kremer, 1982; Kremer et al., 1986) as well as on temperature, affecting the digestion process and thereby the excretion rate (Kremer, 1977; Nemazie et al., 1993). In our case the ammonium release was calculated to be 15 μmol ammonium g DW ctenophore^−1^ day^−1^ (assuming 10 mm oral-aboral length equals 0.04 g DW; Friis-Möller unpublished data), which is within the ammonium release rates commonly found for gelatinous zooplankton (reviewed by Schneider, 1990). Phosphate-levels were also higher in *M. leidyi* treatments than in control treatments (one factor ANOVA *F*_(1, 8)_ = 21.8, *p* = 0.0016) but for nitrite/nitrate no difference were seen (one factor ANOVA *F*_(1, 8)_ = 0.36, *p* = 0.563).

**Table 1:** **Concentrations of inorganic nitrogen and phosphorus (μmol L^−1^) at the start of the experiment and at the end in the different treatments**.

	**NH^+^_4_**	**NO^−^_2_ + NO^−^_3_**	**PO^3−^_4_**
Start water	1.5	<0.1	0.5
Control water	0.78	3.21	1.32
	0.67	3.09	1.06
Water with dipped *M. leidyi*	0.55	3.00	0.82
	0.43	3.17	1.15
Water with copepods	0.90	3.30	0.98
	0.88	3.23	0.69
Water with fed *M. leidyi*	7.96	3.20	2.30
	15.18	3.21	2.44
Water with starved *M. leidyi*	13.17	3.22	1.84
	13.84	3.17	1.44

**Figure 1:**
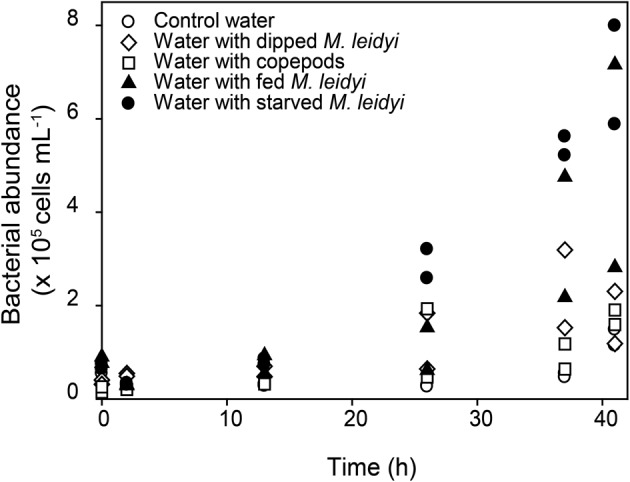
**Changes in bacterial abundance over time**. White symbols represent control treatments and black symbols represent treatments with *M. leidyi*. Data from both replicates are shown for all treatments.

Similar to the ammonium and phosphate levels, bacterial abundance was higher in the *M. leidyi* treatments after 37 h of incubation compared to control treatments (one factor ANOVA *F*_(4, 15)_ = 12.41, *p* = 0.0001). With the SNK post-hoc test the bacterial abundance was seen to differ between starved and fed *M. leidyi* but there were no differences between the water treatment control, the treatment with dipped *M. leidyi* or the treatment with copepods (Figure 1).

The observed responses were anticipated since ctenophores are known to release large amounts of ammonium (Pitt et al., 2009; and references therein). In addition, in combination with the release of bioavailable carbon (Condon et al., 2011), a stimulated bacterial growth was expected (Church, 2008). Interestingly, the observation of extensive bacterioplankton growth associated with both fed and starved *M. leidyi* points to an important role for carbon and nutrients released directly from *M. leidyi* tissue.

### Bacterial community composition

Using 454-pyrosequencing we sought to test the hypotheses that (1) presence and activity of *M. leidyi* stimulate specific bacteria taxa, and (2) that bacteria attached to *M. leidyi* tissue or in the gut differ from those in the surrounding water. A total of 32,736 partial 16S rRNA gene sequences remained after quality controls, yielding on average 4022 reads per sample (range 1420–7770 reads) and a total of 364 unique OTUs in the whole dataset. The rarefaction curves showed saturation for all samples, except for the start water and for the *M. leidyi* gut (data not shown). Hence, our sequencing effort did not cover bacterial diversity well in these two samples.

**Figure 2:**
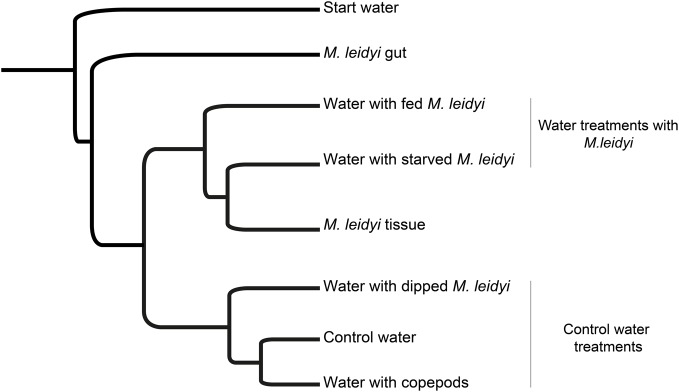
**UPGMA tree calculated from the Jackknife-weighted UniFrac distance matrix, displaying the phylogenetic distances between samples (based on the subsampled datasets of 1043 sequences)**.

Comparisons based on weighted-UniFrac distances between the sub-sampled datasets showed that bacterial communities in waters with fed and starved *M. leidyi* were relatively similar to the community associated with *M. leidyi* tissue (Figure 2). Likewise, the three control treatments were found in one cluster. Dipping of *M. leidyi* for 1 min had a relatively minor effect on bacterial community composition in the surrounding water indicating that a large pulsed release of bacteria loosely associated with the tissue did not take place. The lack of a community response to the presence of copepods was surprising to us because copepods are often heavily colonized by bacteria that may exchange with surrounding water (Møller et al., 2007), and a high concentration of copepods was used relative to *in situ* conditions. The bacterial communities in the initial water and in the *M. leidyi* gut were very different from the other samples. These clustering patterns indicate that presence and/or activity of *M. leidyi* has a distinct effect on bacterioplankton in the surrounding water.

**Figure 3:**
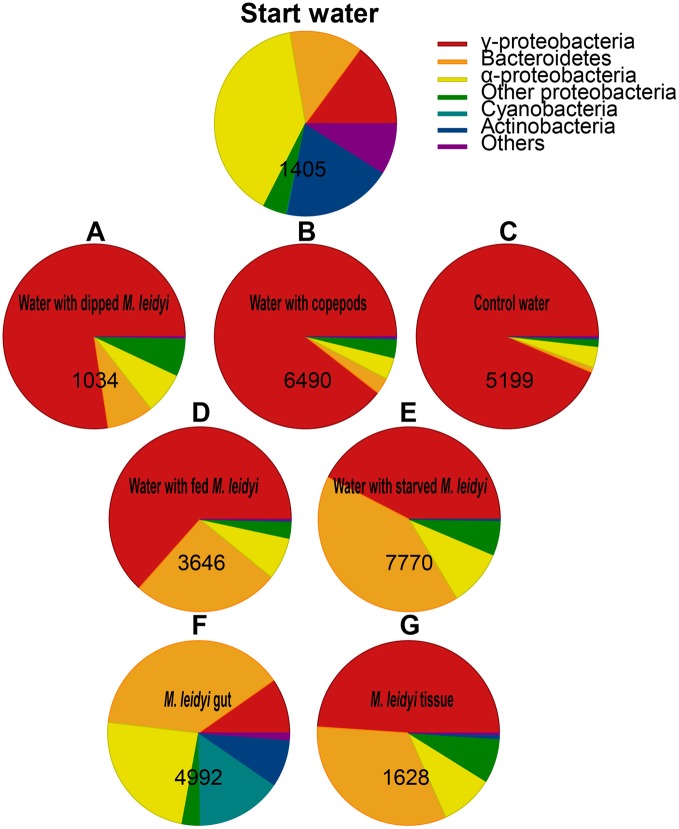
**Relative abundance of the major phyla and proteobacterial sub-classes expressed as the % of total sequences obtained from the sample**. Numbers indicate the total number of sequences per sample. “Others” represent phyla with <1% of relative abundance. Control treatments: water with dipped *M. leidyi* **(A)** water with copepods **(B)** and control water **(C)**. Water treatments with *M. leidyi*: fed **(D)** and starved **(E)**. *M. leidyi* gut **(F)** and tissue **(G)**.

Community composition at the phylum level (phylum and proteobacterial subclass, hereafter referred to as phylum) varied between treatments (Figure 3). The start water was dominated by α-proteobacteria followed by Actinobacteria, γ-proteobacteria, and Bacteroidetes. These phyla are commonly predominant in Skagerrak (Pinhassi et al., 2003; Sjöstedt et al., 2012). After 41 h of incubation, the control treatments were dominated by γ-proteobacteria (Figures 3A–C). Similarly, treatments with starved and fed *M. leidyi* were also dominated by γ-proteobacteria (Figures 3D,E); however, with a much higher contribution of Bacteroidetes (tested in one factor ANOVA *F*_(1, 6)_ = 8.32, *p* = 0.028). Interestingly, proliferation of γ-proteobacteria and Bacteroidetes in the vicinity of *M. leidyi* specimens was also recently demonstrated using fluorescence *in situ* hybridization (Condon et al., 2011). This may indicate niche partitioning defined by the nutrient field in the vicinity of *M. leidyi*; however, the selective drivers causing this bacterial succession remain to be identified.

### Composition of bacteria associated with *M. leidyi* tissue and gut

**Figure 4:**
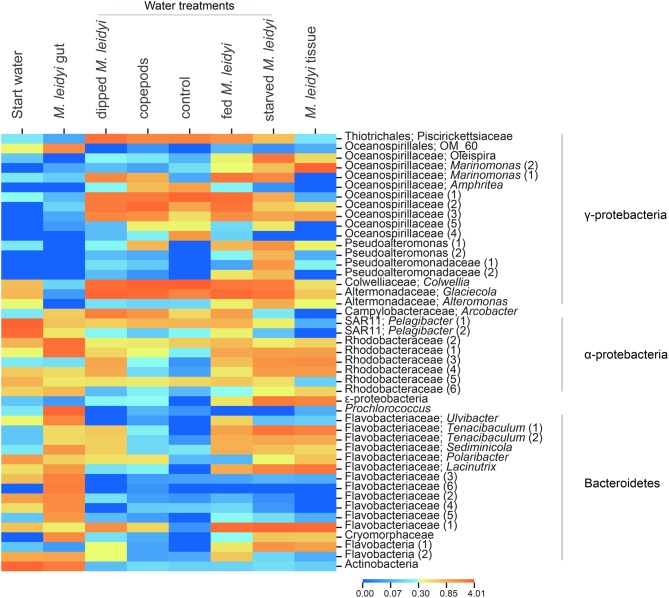
**Heatmap displaying the relative abundances of specific OTUs across the samples**. Only OTUs with a sum of >100 assigned sequences across all samples were used. The color scale represents the log10+1 transformed relative abundance of the number of sequences. Duplicate OTUs were numbered sequentially for clarification.

The gut community was different from the community present on the tissue, and was mainly dominated by Bacteroidetes and α-proteobacteria but also contained Cyanobacteria and Actinobacteria (Figures 3F,G). The dominant OTUs associated with *M. leidyi* (gut, tissue or surrounding water) were related to Flavobacteriaceae (Bacteroidetes) and Rhodobacteraceae (α-proteobacteria); these groups were especially prominent in the gut (Figure 4). *Roseobacter* strains (member of the Rhodobacteraceae family) have been shown to colonize marine algae and dinoflagellates (reviewed in Slightom and Buchan, 2009), and copepods in the North Sea (Møller et al., 2007). This may indicate that some bacterial taxa detected in the gut originate from prey. Based on the approach applied here it is unfortunately not possible to determine to what extent the detected gut sequences originate from bacteria associated with prey, colonizing free-living bacteria, or from a more permanent symbiotic gut microflora. However, since the dissected animal was starved for 24 h prior to dissection, and prey was not visible in the gut, it is likely that the acquired sequences originate from the gut rather than from prey items *per se*.

### Flavobacteriaceae associated with *M. leidyi*

Bacterial community composition associated with *M. leidyi* (water, tissue, and gut) showed a high prevalence of Bacteroidetes (26–41% of the sequences) relative to the control treatments (1–7% of the sequences; tested in one factor ANOVA *F*_(1, 6)_ = 8.32, *p* = 0.028; Figure 3); in particular, members of the Flavobacteriaceae were prominent, especially in the gut (Figure 4). Flavobacteria are known to be enriched on organic particles and show a strong capacity for degrading complex polymers (Kirchman, 2002). Moreover, Flavobacteriaceae produce chitin degrading enzymes and enzymes used for the degradation of algae (reviewed in Bernardet and Nakagawa, 2006) and have recently been reported to play an important role for the degradation of jellyfish tissue (Tinta et al., 2012). Hence, these bacteria conceivably have the metabolic capacity to facility prey digestion in the *M. leidyi* gut as well as to colonize and degrade tissue.

Two main Flavobacterium OTUs were related to the genus *Tenacibaculum* (Figure 4), containing the fish pathogen *Tenacibaculum maritimum*, an agent of the gill disease tenaciaculosis (Handlinger et al., 1997). This bacterium, which has also been reported from two other jellyfish species (Ferguson et al., 2010; Delannoy et al., 2011), produces highly proteolytic enzymes (Bernardet and Nakagawa, 2006) and could play a role in the jellyfish digestive metabolism or, in concert, in degradation of jellyfish tissue. It has been suggested that jellyfish could act as vectors for this pathogen in fish aquacultures (Ferguson et al., 2010; Delannoy et al., 2011). Indeed, the repeated findings, including ours, of the *Tenacibaculum* genus associated with jellyfish is noteworthy; however, additional information on the genetic and functional resemblance of jellyfish associated bacteria and *Tenacibaculum maritimum* is necessary to establish the distribution and ecology of this pathogen in gelatinous plankton.

### Concluding remarks

The present study suggests that *M. leidyi* from the Gullmar fjord in autumn harbors specific bacterial communities in the gut and tissue, and that the presence and activity of *M. leidyi* influences bacterioplankton activity and community composition in the vicinity of the jellyfish. Comparisons of community composition in the individual treatments should, however, be interpreted with caution, since they are based on sequencing of single samples from small bottle incubations. While the bacterial growth in replicate incubations in most cases yield confidence in a corresponding similar compositional succession, extensive sequencing of true replicate samples would be needed to draw firm conclusions on community composition in specific treatments. The high reproducibility of 454 pyrosequencing gives, however, some confidence in the obtained results. For instance, a phylum-level standard deviation of less than 2% of read abundance between technical replicates was recently shown for indigenous bacterial communities in sediments (Pilloni et al., 2012). Finally, the strong selection for Flavobacteriaceae was supported by our data from the gut, tissue, and the treatments with *M. leidyi*. This finding as well as the stimulation of bacterial growth in the vicinity of the ctenophore may have several implications: (1) Due to the specific bacterial communities associated with *M. leidyi*, it may be speculated that it can serve as a vector for relocation of microbial species between pelagic environments, a phenomenon demonstrated for zooplankton (Grossart et al., 2010); (2) Our observations point to strong local bottom-up effects associated with *M. leidyi*. Hence, in addition to food web interactions elicited by jellyfish grazing (Pitt et al., 2009), enhanced bacterioplankton growth in the vicinity of the jellyfish could contribute to the spatial heterogeneity of bacterioplankton growth and community composition in the sea. This could be particularly important during ctenophore blooms (like in autumn in the Gullmar fjord) or in relation to physical gradients (like for example temperature and salinity), physical discontinuities (like fronts) or surface features like Langmuir cell circulations where temporary aggregations of gelatinous plankton occur (Graham et al., 2001). Within such hydrodynamic boundaries, ctenophores, and consequently elevated nutrient levels, could be retained, leading to local changes in bacterioplankton activity and community composition. For instance, a selection for Flavobacteriaceae, with their ability to degrade complex polymers (Kirchman, 2002), could affect the distribution and fluxes of nutrients and carbon, ultimately affecting dynamics of primary production and higher trophic levels.

Along with other recent studies (e.g., Pitt et al., 2009; Condon et al., 2011; Tinta et al., 2012), the present work contributes to the emerging picture of extensive and dynamic interactions between gelatinous plankton (live and dead) and bacteria, which has local consequences for bacterial activity and community composition, and likely influence jellyfish ecology. A deeper mechanistic understanding of the extent and spatio-temporal variability is, however, needed in order to establish the ecological implications of jellyfish—bacteria interactions in marine waters.

### Conflict of interest statement

The authors declare that the research was conducted in the absence of any commercial or financial relationships that could be construed as a potential conflict of interest.
